# Suspected Cat-to-Human Transmission of SARS-CoV-2, Thailand, July–September 2021

**DOI:** 10.3201/eid2807.212605

**Published:** 2022-07

**Authors:** Thanit Sila, Jutapoln Sunghan, Wison Laochareonsuk, Smonrapat Surasombatpattana, Chanon Kongkamol, Thammasin Ingviya, Pisud Siripaitoon, Narongdet Kositpantawong, Siripen Kanchanasuwan, Thanaporn Hortiwakul, Boonsri Charernmak, Ozioma Forstinus Nwabor, Kachornsakdi Silpapojakul, Sarunyou Chusri

**Affiliations:** Prince of Songkla University Faculty of Medicine, Songkhla, Thailand (T. Sila, W. Laochareonsuk, S. Surasombatpattana, C. Kongkamol, T. Ingviya, P. Siripaitoon, N. Kositpantawong, S. Kanchanasuwan, T. Hortiwakul, B. Charernmak, O.F. Nwabor, K. Silpapojakul, S. Chusri);; Prince of Songkla University Faculty of Veterinary Science, Songkhla (J. Sunghan)

**Keywords:** COVID-19, coronavirus disease, SARS-CoV-2, severe acute respiratory syndrome coronavirus 2, viruses, respiratory infections, zoonoses, vaccine-preventable diseases, cat, feline, Thailand

## Abstract

A veterinarian in Thailand was diagnosed with COVID-19 after being sneezed on by an infected cat owned by an infected patient. Genetic study supported the hypothesis of SARS-CoV-2 transmission from the owner to the cat, and then from the cat to the veterinarian.

COVID-19, caused by SARS-CoV-2, has been suspected to be a zoonosis because of its link to a live animal market in Wuhan, China (*1*). In addition, several countries in the Americas, Africa, Europe, and Asia have reported the occurrence of COVID-19 in various animal species, including minks, cats, dogs, lions, and tigers (*2*). However, most of these infections primarily originated from humans and were transmitted to the animal (i.e., reverse zoonosis), with numerous reports in domestic cats (*2*,*3*). A recent report describes a possible animal-to-human transmission of SARS-CoV-2 from infected farm minks to farmworkers in the Netherlands (*4*). We describe a suspected zoonotic SARS-CoV-2 transmission from a cat to a human.

## Case Report

During July–September 2021, the COVID-19 pandemic was shifting from the Alpha variant to the Delta variant. On August 15, 2021, in Songkhla, a business province in southern Thailand, patient A, a 32-year-old previously healthy female veterinarian who lived alone in a dormitory on campus visited the hospital of Prince of Songkla University, located in Hatyai District, Songkhla Province, with a history of fever, clear nasal discharge, and productive cough of 2 days’ duration. Results of a physical examination, including a chest radiograph, were otherwise unremarkable. When questioned about her history, she said that 5 days earlier, she and 2 other veterinarians (patients E and F) had examined a cat belonging to 2 men (patients B and C).

**Figure 1:**
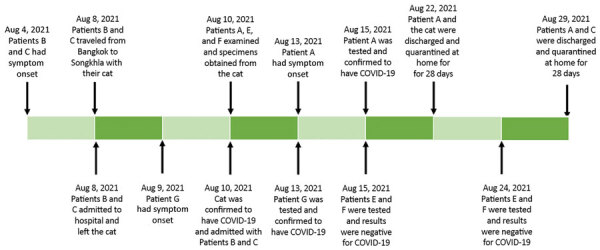
Timeline of suspected cat-to-human transmission of SARS-CoV-2, Thailand, August 2021.

Patients B and C, a 32- and 64-year-old son and father, were from Bangkok, the capital city of Thailand. They were confirmed positive for SARS-CoV-2 infection by reverse transcription PCR (RT-PCR) a day earlier and were transferred to Prince of Songkla University hospital because of the unavailability of hospital beds in Bangkok. Together with their cat, patients B and C were transported by an ambulance in a 20-hour, 900-km trip on August 8, 2021 (Figure 1). On arrival, the patients were immediately admitted to an isolation ward. The cat that had been sleeping on the same beds as the patients was sent to the university veterinarian hospital for an examination by patient A on August 10, 2021, and found to be clinically normal. Patient A retrieved nasal and rectal swab specimens from the cat while patients E and F restrained it. During the nasal swabbing, the sedated cat sneezed in the face of patient A. All 3 veterinarians were wearing disposable gloves and N95 respirator masks without face shields or eye goggles at the time. The entire veterinarian–cat encounter lasted ≈10 minutes.

Three days after exposure to the cat, patient A became symptomatic but did not seek medical consultation until August 15, when the RT-PCR test results of the cat returned COVID-19–positive (Table). On investigation, nasopharyngeal swab specimens from patient A showed detectable SARS-CoV-2 (Table). Patients A, B and C and the cat were admitted for isolation in the hospital. Test results for the swab specimens from patients E and F were negative.

**Table:** Sample metadata of SARS-CoV-2 genome derived from feline and human patients after suspected cat-to-human transmission of SARS-CoV-2, Thailand, July–September 2021*

Patient	Sequence ID	Type of sample	Cycle threshold		PANGO lineage	Pairwise distance, bp
			ORF1ab gene	N gene		
A	Patient_A	Nasopharyngeal swab	20	16	B.1.167.2	0.00
B	Patient_B	Nasopharyngeal swab	19	22	B.1.167.2	0.00
C	Patient_C	Nasopharyngeal swab	15	20	B.1.167.2	0.00
G	Patient_G	Nasopharyngeal swab	24	26	B.1.167.2.30	40.00
Cat	Throat_cat	Throat swab	17	16	B.1.167.2	0.00
Cat	Rectal_cat	Rectal swab	21	15	B.1.167.2	0.00

*PANGO lineages identified by using Pangolin and Pangolearn (https://cov-lineages.org/resources/pangolin/pangolearn.html). N, nucleoprotein; ORF, open reading frame.

No close contacts of patient A were diagnosed with COVID-19. Contact tracing investigations of all the 30 personnel working at the Veterinary Hospital identified 1 additional contact with COVID-19, a veterinarian who worked in the Department of Large Animals (patient G). Patient G had fever onset 1 day before the cat’s arrival and had tested positive for COVID-19 on August 13, 2021. He reported no direct or indirect contact with the cat or patients A, E, or F.

**Figure 2:**
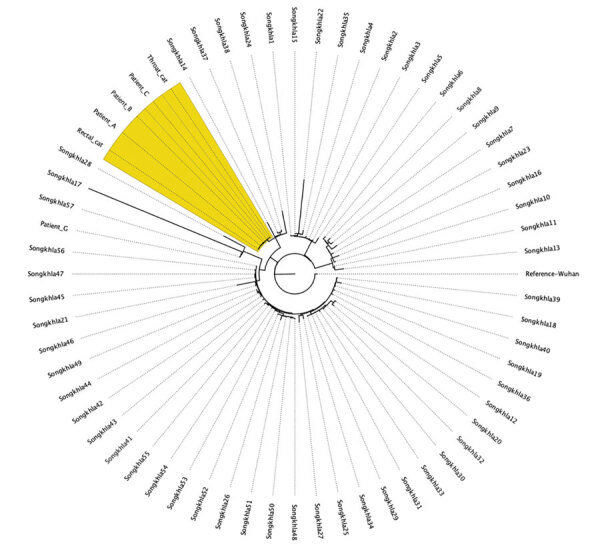
Phylogenetic tree of SARS-CoV-2 genome sequences retrieved from patients A, B, and C and the cat belonging to patients B and C (yellow shading) compared with reference sequences from COVID-19 patients from Songkhla Province, Thailand, July–September 2021. Tree constructed with IQ-TREE (http://www.iqtree.org) by using the maximum-likelihood method and 1,000 bootstrap replicates.

Before genotyping, we tested viral RNA from the cat, patients A, B, C, and G, and other patients in Songkhla Province for SARS-CoV-2 by using RT-PCR. Primer sets were designed to target the nucleoprotein and open reading frame 1ab genes (Table). For viral whole-genome sequencing, we performed library preparation for the SARS-CoV-2 genome by using QIAseq DIRECT SARS-CoV-2 kits (QIAGEN, https://www.qiagen.com) on an Illumina NextSeq 550 (Illumina, https://www.illumina.com) at the Translational Medicine Research Center at Prince of Songkla University. We identified the PANGO lineages by using Pangolin and Pangolearn (https://cov-lineages.org/resources/pangolin/pangolearn.html) (Table). We generated maximum-likelihood phylogenetic trees of the representative cases and others from aligned consensus sequences by using an IQ-TREE (http://www.iqtree.org) with 1,000 bootstraps. We visualized the phylogenetic tree by using FigTree (http://tree.bio.ed.ac.uk/software/figtree). The genomes of patients B and C and the cat were identical to that obtained from patient A, but they were distinct from those of other patients in the same province (Figure 2). The pairwise distance between patient A and the cat was shown to be similar (*5*) by MEGA 11 (https://www.megasoftware.net) with 1,000 bootstraps (Table).

## Conclusions

The identical SARS-CoV-2 genome sequences obtained from patient A and the sequences derived from the cat and its 2 owners, together with the temporal overlapping of the animal and human infections, indicated that their infections were epidemiologically related. Because patient A had no prior meetings with patients B or C, she probably acquired SARS-CoV-2 from the cat when it sneezed in her face. The genome sequences were distinct from that of patient G and other sequences circulating in the same province, and by using the pairwise distance formula, we were able to rule out external transmission (*5*). The Alpha variant was widely spread until the end of July 2021 in Songkhla Province; on the other hand, in Bangkok, the Delta variant has been widespread since the beginning of July 2021 (*6*).

The transmission chain of SARS-CoV-2 infections in this cluster probably began in Bangkok. Cats are known to be susceptible to SARS-CoV-2 infection (*8*–*10*), especially during close interactions with humans with symptomatic SARS-CoV-2 infections (*7*). Because infected cats have relatively short incubation and contagious periods (*8*–*10*), this cat probably had acquired its SARS-CoV-2 infection no longer than a week before possibly transmitting the disease to patient A.

Although direct or indirect (fomites) contacts are also potential routes of transmission to patient A, these possibilities are less likely because she wore gloves and washed her hands before and after examining the cat. Transmission from the cat sneeze is hypothesized because of this brief but very close encounter. The relatively low RT-PCR cycle thresholds (*11*) in the nasal swab obtained from the cat suggest that the viral load was high and infectious (*12*,*13*). Because patient A wore an N95 mask without a face shield or goggles, her exposed ocular surface was vulnerable to infection by droplets expelled from the cat. Her infection signifies the possibility of ocular transmission and the importance of wearing protective goggles or face shields in addition to a mask during close-range interactions with high-risk humans or animals.

In summary, we have provided evidence that cats can transmit the SARS-CoV-2 infection to humans. However, the incidence of this transmission method is relatively uncommon because of the short (median 5 days) duration of cats shedding viable viruses (*8*–*10*). Nevertheless, to prevent transmission of SARS-CoV-2 from humans to cat, persons with suspected or confirmed COVID-19 should refrain from contact with their cat. Eye protection as part of the standard personal protection is advisable for caregivers during close interactions with cats suspected to be infected.
